# Negative impact of hippocampal radiotherapy dose on memory function in patients with brain metastases

**DOI:** 10.1093/nop/npag001

**Published:** 2026-01-10

**Authors:** Eva E van Grinsven, Fia Cialdella, Marielle E P Philippens, Joost J C Verhoeff, Szabolcs David, Martine J E van Zandvoort

**Affiliations:** Department of Neurology & Neurosurgery, University Medical Center Utrecht, Utrecht, The Netherlands (E.E.V.G., M.J.E.V.Z.); Department of Experimental Psychology and Helmholtz Institute, Utrecht University, Utrecht, The Netherlands (M.J.E.V.Z.); Department of Radiation Oncology, University Medical Center Utrecht, Utrecht, The Netherlands (F.C., M.E.P.P., J.J.C.V., S.D.); Department of Medical Oncology, University Medical Center Utrecht, Utrecht, The Netherlands (F.C.); Department of Radiation Oncology, University Medical Center Utrecht, Utrecht, The Netherlands (F.C., M.E.P.P., J.J.C.V., S.D.); Department of Radiation Oncology, University Medical Center Utrecht, Utrecht, The Netherlands (F.C., M.E.P.P., J.J.C.V., S.D.); Department of Radiation Oncology, Amsterdam University Medical Center, Amsterdam, The Netherlands (J.J.C.V., S.D.); Department of Radiation Oncology, University Medical Center Utrecht, Utrecht, The Netherlands (F.C., M.E.P.P., J.J.C.V., S.D.); Department of Radiation Oncology, Amsterdam University Medical Center, Amsterdam, The Netherlands (J.J.C.V., S.D.); Department of Neurology & Neurosurgery, University Medical Center Utrecht, Utrecht, The Netherlands (E.E.V.G., M.J.E.V.Z.); Department of Experimental Psychology and Helmholtz Institute, Utrecht University, Utrecht, The Netherlands (M.J.E.V.Z.)

## Abstract

**Background:**

Brain metastases (BMs) are often treated with stereotactic radiosurgery (SRS). Even with the high dose fall-off at tumor borders, some dose is delivered outside the BMs to ensure adequate tumor coverage. This may include radiation to the neurogenic niches (ie, subventricular zones [SVZ] and hippocampi), which possibly play a role in neurocognitive functioning (NCF). In this study, we assessed the impact of radiotherapy dose on the SVZ and hippocampi compared with changes in NCF 3 months post-SRS, focusing on memory and information processing speed (IPS) in adult patients with BMs.

**Methods:**

Neurocognitive assessments were performed before and 3 months post-SRS, and reliable change indices were calculated to assess NCF changes. The SVZ and hippocampal regions were delineated on pre-SRS T1-weighted MRI images and median radiotherapy dose per region was calculated.

**Results:**

Multivariable linear regression analyses in 36 patients corrected for age, previous brain radiotherapy, number of BMs, and previous systemic therapy indicated a significant association between higher hippocampal radiotherapy dose and decline in memory, specifically in Hopkins Verbal Learning Test-Revised immediate recall, delayed recall, and Rey Osterieth Complex Figure Test delayed copy (all *P* < .05). Conversely, no significant association was found between dose to the SVZ and memory, nor between dose to either region and IPS.

**Conclusions:**

These results suggest potential benefits of minimizing radiation exposure to the hippocampi, even with SRS, to help preserve memory in patients with BMs. Further prospective evaluation is warranted to validate these findings and inform clinical decision-making regarding radiotherapy protocols for preserving NCF in this patient population.

Key Points

- Higher hippocampal dose is correlated with declined memory performance 3 months post-SRS
- No association between SVZ dose and change in NCF 3 months post-SRS
- Avoiding irradiation in hippocampus during SRS could be essential for preserving memory functions

Importance of the Study

This investigation explores the impact of radiation on the subventricular zones (SVZ) and hippocampi, on neurocognitive functioning of patients with brain metastases (BMs). Building on prior research primarily focused on patients with glioma, our investigation reveals a significant link between higher hippocampal radiation doses and memory decline 3 months post-SRS. Specifically, this was found for verbal learning (Hopkins Verbal Learning Test-Revised [HVLT-R] immediate) and retrieval (HVLT-R delayed recall), as well as incidental memory (Rey Osterieth Complex Figure Test delayed). Conversely, no significant correlation was found between radiation dose to the SVZ and memory decline, nor between dose to either region and changes in information processing speed 3 months post-SRS. These findings highlight radiation’s impact on hippocampi should be considered when developing treatment strategies for patients with BMs. Further prospective studies using elaborate cognitive testing are necessary to confirm these results and guide decisions on radiotherapy protocols that aim to preserve neurocognitive function in this patient group.

Patients with brain metastases (BMs) represent a rapidly growing population, as BMs affect roughly 10% to 30% of adults diagnosed with cancer.^1^ This number has increased in recent years due to more sensitive detection methods for BMs, longer life expectancy of cancer patients due to improved systemic therapy for the primary tumor, and an aging population being more prone to develop cancer.^2^ BMs are most commonly treated by either local radiotherapy (RT) or a combined treatment of surgical resection followed by RT, although in selected cases systemic therapy with intracranial activity alone may be considered. The aim of local therapy is to improve overall survival (OS) and to alleviate symptomatic progression of the BMs.^3^ Due to risk of early and late toxicity, stereotactic radiosurgery (SRS) is preferred over whole-brain radiotherapy (WBRT), especially for a limited number of BMs (eg, less than 10).^4-6^ While previous research has predominantly focused on the cognitive and neurological late effects of WBRT, SRS has emerged as the recommended treatment modality for this patient group based on consensus guidelines. Nonetheless, cranial RT may still induce damage to healthy or healthy-appearing brain tissue, which may be linked to the declined cognitive performance.^7-12^

Previous research reports substantial variety in this radiation-induced cognitive decline, both regarding the affected cognitive domains and the severity of the cognitive deficits. On an individual level, approximately 40% of patients with BMs show a decline in cognitive performance in the 9 months following SRS across multiple cognitive domains.^13^^,^^14^ This comes on top of the cognitive impairment half of the patients already experience due to the tumor and treatments prior to brain RT,^15-17^ illustrating their cognitive vulnerability. Multiple cognitive domains can be affected after radiotherapy, but memory performance seems to be especially vulnerable both before and 3 months after radiotherapy and information processing speed often declining after 3 months.^10^^,^^17^ The mechanisms underlying this cognitive decline are most likely complex and multifactorial, including vascular damage, neuroinflammation, altered neuronal function, and neurogenesis impairment.^18^

Even with high dose fall-off at tumor borders, such as in SRS, some radiotherapy dose is delivered outside the BMs to ensure adequate tumor coverage. This is due to the planning target volume (PTV) concept used in radiotherapy and dose planning techniques. Patients receiving radiation therapy encompassing neural stem cells (NSCs) areas may be particularly vulnerable for radiation-induced brain damage as NSCs are presumed to play a crucial role in the brain’s repair mechanisms.^19^ Neural stem cells are found within specialized areas known as *neurogenic niches*, which involve 2 distinct regions in the adult brain: (1) the subventricular zone (SVZ), located underneath the ependymal layer of cells that line the lateral ventricles and (2) the subgranular zone, part of the hippocampal dentate gyrus.^20^ Neural stem cells induce neurogenesis, which is the process of generating new neurons from NSCs. This process enables the replacement of lost neurons caused by brain injuries such as tumor growth, surgical procedures, or radiation exposure.^21^ In addition, adult NSCs, particularly those concentrated in the hippocampus, actively contribute to the regulation of various cognitive functions, highlighting their importance in cognitive processes.^22^

Irradiation of the neurogenic niches is hypothesized to play a role in reducing adult neurogenesis in 2 ways: (1) inducing acute apoptosis in dividing cells and (2) reducing the production of new neurons by decreasing the pool of mitotic NSCs.^23^ Two recent studies by our group have shown worse OS in both patients with glioma and BMs after irradiation of the neurogenic niches.^24^^,^^25^ Based on these findings, avoiding irradiation of the neurogenic niches may result in better OS. However, with an already prolonged life expectancy in patients with BMs, patient-centered care is increasingly focused on not only extending the life span, but especially on maintaining or even improving the quality of life (QoL). Accordingly, the importance of preserving neurocognitive function by minimizing radiation exposure to the hippocampus has been established in previous studies.^26-28^ There are only few studies reporting on the impact of SVZ radiation dose on neurocognitive decline in brain tumor patients. A recent prospective study on patients with glioma demonstrated a significant correlation between cognitive decline and increased radiation doses specifically directed at the SVZ.^29^ This highlights the importance of strategic radiotherapy dose planning in preserving cognitive functioning.

Although the hippocampus is known to be radiosensitive and rarely harbors BMs, little is known about the relationship between hippocampal and SVZ radiation dose and cognitive outcomes in the context of SRS. In contrast to existing studies on hippocampal-avoidance whole-brain radiotherapy (HA-WBRT), the present study examines continuous dose-response relationships in SRS, where dose fall-off is steep and overall brain exposure is minimal. This approach also allows us to consider whether damage to hippocampal connections, rather than the hippocampus alone, may contribute to posttreatment cognitive decline, thereby providing novel insights for optimizing radiotherapy to preserve cognitive function.

Building upon these considerations, the current study aims to further improve our understanding of radiation-induced cognitive decline by investigating the effect of radiation on the neurogenic niches on cognitive decline 3 months after SRS in a cohort of patients with BMs. In the current study, we have focused on memory performance and information processing speed. Both domains have previously shown to be vulnerable to cognitive decline after radiotherapy, as shown in a subset of our study population. Furthermore, considering the established association between episodic memory and the hippocampus^30^ contrasted with the more widely distributed neural network underlying processing speed,^31^^,^^32^ our focus enables an assessment of the specificity of hippocampal-related cognitive changes. This combination of empirically vulnerable and theoretically distinct cognitive domains allows for a more precise evaluation of radiation effects on neurogenic niches. The findings could provide further evidence supporting the avoidance of these neurogenic niches, which has been demonstrated to be technically feasible.^33^

## Materials and Methods

### Study Set-up and Population

Data were prospectively collected from the Cohort for patient-reported Outcomes, Imaging and trial inclusion in Metastatic BRAin disease (COIMBRA, NCT05267158) and the Assessing and Predicting Radiation Influence on Cognitive Outcome using the cerebrovascular stress Test (APRICOT) study, as described previously.^10^^,^^17^^,^^34^ In short, the study population consisted of adult patients (≥18 years) with either radiographic and/or histologic proof of metastatic brain disease referred to the University Medical Center Utrecht (UMCU) for brain radiotherapy. Patients were noneligible if they were unable to understand the Dutch language or had developmental, psychiatric, or cognitive disorders prior to radiotherapy that hindered the patients’ understanding of the informed consent procedure. For both studies, neurocognitive assessments (NCAs) were performed before and 3 months after SRS. Both studies were performed in accordance with the Declaration of Helsinki,^35^ and the UMCU institutional ethical review approved both the COIMBRA and APRICOT study (#18-642 and #18-747, respectively). Written informed consent was obtained from all participants prior to participation. For the current study, only patients with completed NCA before and 3 months after completion of RT were analyzed. Patients who had received additional cranial radiotherapy prior to the 3-month NCA were excluded.

### Data Collection

#### Clinical data

Patient characteristics and clinical information were obtained from the semi-structured interview before the NCA and from the electronic health record (HiX, Chipsoft, the Netherlands). These data included sex, age at inclusion, level of education according to the Verhage criteria^36^ (see Table S1 for detailed classification), handedness, Karnofsky Performance Status (KPS),^37^ primary tumor origin, presence of extracranial metastases, prior systemic therapies (ie, chemotherapy, immunotherapy, and targeted therapy), number and location of BMs, and symptoms at BM diagnosis. All patients were treated with single fraction SRS. Gross total volume (GTV) and PTV were derived from clinical datasets.

#### Neurocognitive assessment

For both the COIMBRA and APRICOT studies, a comprehensive NCA was used to assess objective cognitive performance. All tests are internationally widely used, standardized psychometric instruments for assessing neurocognitive deficits in the major neurocognitive domains, as described previously.^10^^,^^17^^,^^34^ To minimize practice-effects, parallel versions were used at follow-up testing when available. All NCAs were performed in-person by trained personnel. The NCA was planned to be completed within approximately 90 minutes.

While neuropsychological tests often tap into more than 1 neurocognitive domain, tests were classified into different neurocognitive domains based on the available literature and clinical experience. Several components of episodic memory were measured using the Hopkins Verbal Learning Test-Revised (HVLT-R; verbal learning, verbal delayed memory, verbal recognition memory), the delayed copy of the Rey Osterieth Complex Figure Test (ROCFT; incidental nonverbal delayed memory), and the immediate recall of the Visual Association Test (VAT; associative memory). For processing speed, the performance on the Trail Making Test (TMT) part A, and Stroop D-KEFS version condition I (naming speed), and II (reading speed) was considered.

For this study, cognitive data (specifically regarding memory and information processing speed) acquired from October 2020 to April 2023 were used. Each neuropsychological test was scored according to standardized scoring criteria. The uncorrected scores were transformed into *z*-scores based on the mean and standard deviation of control populations derived from published norm data. Scores were corrected for age and education where appropriate. Neurocognitive impairment per domain was defined as a *z*-score ≤−1.5 on any of the administered tests within the domain.

Individual change in neurocognitive performance 3 months after radiotherapy was assessed and classified using the RCI as formulated by Jacobson and Truax.^38^^, ^^39^ This RCI accounts for the test-retest reliability of the task. The assumption of normal distribution for RCIs permits the evaluation of the statistical significance of the change. In the current study, an α level of 0.10 was used. Thereby, RCI values ≥1.645 present improvement, ≤−1.645 present decline, and values not exceeding ±1.645 indicate stable cognitive performance on the task. Although RCI values indicate statistically reliable change beyond measurement error, these changes do not necessarily correspond to crossings of clinical impairment thresholds (eg, *z*-score ≤−1.5). Therefore, a patient may show reliable decline on the RCI measure while still performing within a nonimpaired range according to conventional neuropsychological cut-offs. For illustrative purposes, change in neurocognitive performance per domain was defined as “declined”/“improved” if at least 1 task showed decline/improvement and performance on other tasks remained stable, as “stable” when all tasks indicated stable cognitive performance and as “mixed” when at least 1 task showed declined and another improved performance.

### Image Acquisition and Processing

**Figure 1.**
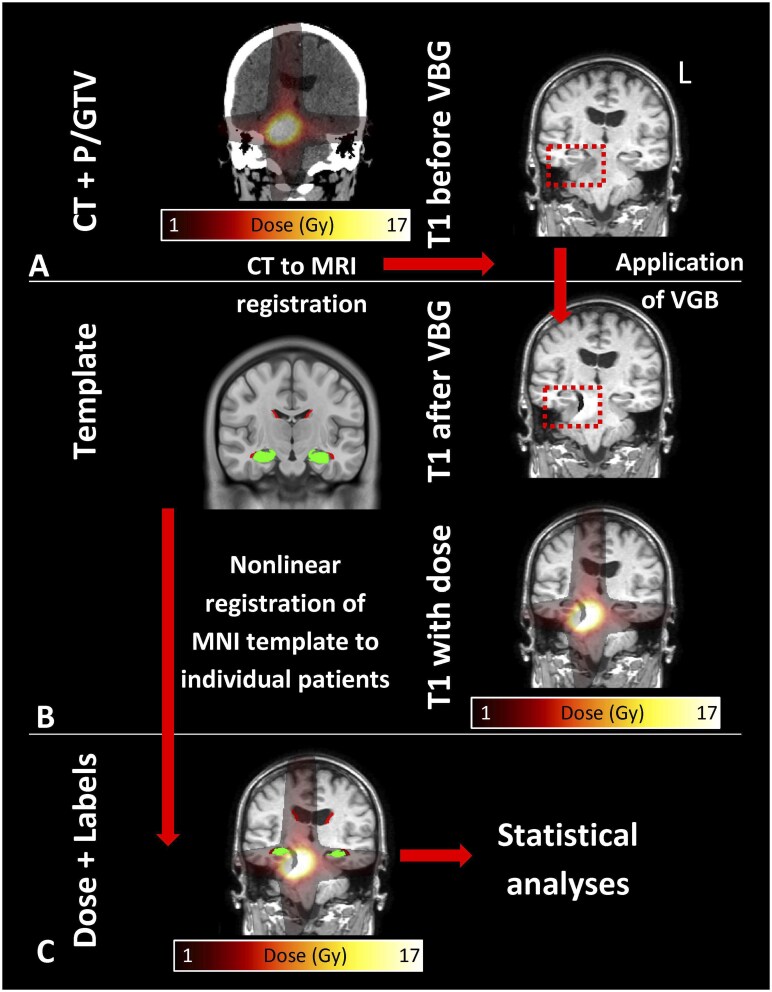
Image processing pipeline. Panel A shows an example patient’s planning CT and dose distribution on the left and the same patients planning MRI is shown on the right. The CT image is linearly (rigidly) registered to the planning MRI. Panel B shows the VBG-enhanced T1 with and without the dose distribution on the right. Panel B left shows the brain template with the SVZ regions (red) and hippocampi (green) which are registered to the VBG-enhanced T1 MRI. Panel C shows the result of the image processing: dose can be calculated within each SVZ and hippocampus areas. Radiological view: left is right. Abbreviations: gross total volume; PTV, planning target volume; SVZ, subventricular zone; VBG, virtual brain grafting.

The details of image acquisition and processing are extensively covered in our previous works.^24^^,^^25^ In summary, we used the pretreatment planning MRIs, CTs, dose maps and GTVs and PTVs delineated by radiation oncologists, which were acquired as part of the standard clinical care. Subventricular zone^24^ and hippocampus^40^ labels were acquired via nonlinear atlas registration using CAT12.^41^ To aid the image processing, before using CAT12, all T1 MRIs were preprocessed with virtual brain grafting (VBG).^42^ During VBG, all abnormal or abnormal-looking tissue such as BMs, edema, but also tumor cavity, is replaced with healthy-appearing but fake brain tissue, while the rest of the brain is untouched. This fake tissue enables CAT12 to perform high-quality atlas registration. Figure 1 shows a visual overview of the image processing pipeline in an example patient. First, the patients’ T1 MRIs are processed with VBG. The enhanced images are then processed with CAT12, which includes a nonlinear registration of a brain template (MNI) along with template space atlas labels of the SVZ and hippocampus. After this step, all required information is in the same anatomical space: radiation dose and the atlas labels for each patient. Next, we calculated the dose statistics per SVZ and hippocampus labels for subsequent statistical analyses. Labels covering the fake tissue were excluded from the analysis and were used only to facilitate the atlas registration process. Consequently, only the nonaffected parts of SVZ and hippocampi were used in the analyses. Subsequently, we calculated the mean dose per atlas label. Virtual brain grafting was applied for every patient, as in our experience even when the tissue abnormality is spatially far from the SVZ or the hippocampi, for example in the cerebellum, it still improves the quality of the atlas registration overall and is thus beneficial for the subsequent analyses.

### Statistical Analyses

In this study, we utilized univariable and multivariable linear regression analyses to investigate the impact of radiation dose in the neurogenic niches (SVZ/hippocampus) on change in cognition (memory/processing speed) in patients with BMs. To be able to assess the relationship between changes in neurocognitive functioning and dose per neurogenic region, the change (ie, RCI-score) per test was considered in the analysis. Covariates included age at baseline, previous brain RT, previous systemic treatment, number of BMs treated with current SRS, and volume of SVZ/hippocampus. Previous systemic therapy was included as a binary variable (yes/no) to control for possible confounding effects of prior systemic treatment. Analyses were deemed statistically significant at *P* < .05 and were not corrected for multiple comparisons due to the exploratory nature of this study. Furthermore, we used the variance inflation factor (VIF) to evaluate the presence of multicollinearity among all covariates for each independent variable. VIF > 5 was seen as multicollinearity. Differences between patients completing and not completing the follow-up NCAs were assessed using χ^2^ test for categorical data and Mann-Whitney *U* tests for continuous data. All statistical analyses were performed using IBM SPSS (for Windows, version 25.0.0).

## Results

### Participants

In total, 71 patients performed the preradiotherapy NCA between October 2020 and April 2023. In total, 35 patients were lost to follow-up for NCA, most often due to worsening medical conditions (15/35) or death (8/35) (Figure S1). Importantly, only 2 patients were excluded because they received additional cranial radiotherapy between baseline and the 3-month follow-up. Thereby, 36 patients (16 women) completed the 3-month follow-up NCA and were included in the current analysis. Patients who did not complete the 3-month follow-up had a lower KPS than patients who did complete the 3 months follow-up (*P* = .001). None of the other characteristics as shown in Table 1 significantly differed between patient groups. The median age of the included patients was 63 years old (interquartile range [IQR] = 56.3-71.3) and median time between preradiotherapy and postradiotherapy NCA was 16 weeks (IQR = 14.0-16.0). Most patients (42%) had 2 to 4 BMs based on the pre-SRS MRI. Brain metastases most often originated from lung cancer (47%) or melanoma (27%).

**Table 1.** Preradiotherapy sociodemographic and clinical characteristics of the patient population

	BMs patients (n = 36)
Age, years, median (IQR)	63 (56.3-71.3)
Sex (female), n (%)	16 (44.4)
Educational level,^a^ n (%)	
3	2 (5.6)
4	7 (19.4)
5	11 (30.6)
6	10 (27.8)
7	6 (16.7)
KPS, median (IQR)	80 (80-90)
KPS ≥90, n (%)	15 (41.6)
No. of BMs, n (%)	
1	12 (33.3)
2-4	15 (41.7)
5-10	7 (19.4)
>10	2 (5.6)
Median total GTV-volume in cc (IQR)	6018 (2264-15 767)
100%- PTV Dose on largest brain metastasis, n (%)	
1x15 Gy	3 (8.3)
1x16 Gy	1 (2.8)
1x18 Gy	8 (22.2)
1x21 Gy	16 (44.4)
1x24 Gy	8 (22.2)
Subventricular zone-tumor any contact, n (%)	8 (22.2)
Hippocampus-tumor any contact, n (%)	2 (5.6)
Subventricular zone-volume, median (IQR)	9854 (8457-11 385)
Hippocampi-volume, median (IQR)	7331 (6772-7781)
Dose in SVZ in Gy, median (IQR)	1.12 (0.19-2.04)
Dose in hippocampi in Gy, median (IQR)	0.89 (0.14-1.88)
Primary tumor origin, n (%)	
Lung cancer	16 (44.4)
Melanoma	10 (27.8)
Breast cancer	3 (8.3)
Renal cell carcinoma	3 (8.3)
Other	4 (11.2)
Extracranial metastases, n (%)	23 (63.9)
Previous brain radiotherapy, n (%)	5 (13.9)
Previous BMs resection, n (%)	8 (22.2)
Extent of surgery	
Partial resection, n (%)	4 (50)
Complete resection, n (%)	4 (50)
Previous systemic therapy, n (%)	27 (75)
Symptomatic BMs at diagnosis, n (%)	23 (63.9)

^a ^According to Verhage classification.^36^ Abbreviations: BMs, brain metastases; GTV, gross total volume; IQR, interquartile range; KPS, Karnofsky Performance Scale; PTV, planning target volume; SRS, stereotactic radiosurgery; SVZ, subventricular zone.

### Linear Regression Analysis

An elaborate review of both the preradiotherapy and postradiotherapy cognitive functioning of a subset of this patient population has been described elsewhere.^10^ The data utilized for the present analysis are illustrated in Figure S2, revealing considerable fluctuations in post-SRS changes in cognitive functioning across patients, including both substantial improvements and declines. As mentioned, a significant decrease in cognitive performance 3 months after SRS (ie, RCI ≤ −1.645) was not always mirrored by impaired cognitive performance (ie, *z*-score ≤ −1.5) at the same time point. In other words, some patients exhibited noticeable cognitive decline post-SRS, but still performed above the threshold for cognitive impairment.

**Figure 2.**
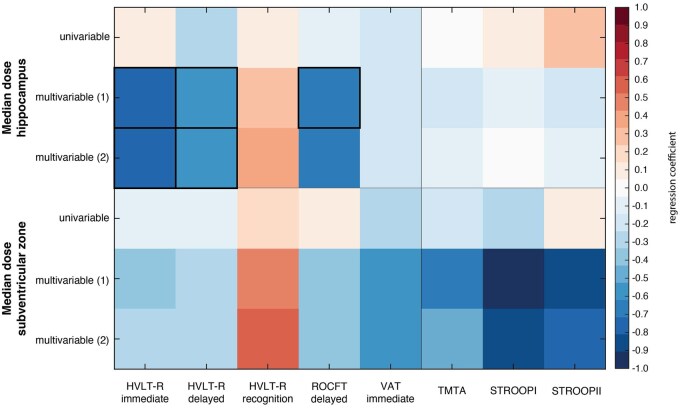
Visual overview of the regression coefficients of the univariable and both multivariable linear regression analyses for all memory and information processing speed tests for median radiotherapy dose on the hippocampi and the subventricular zone. Blue colors indicate a negative relationship and red values a positive relationship. Bold outlined blocks indicate statistically significant relationships. Multivariable analyses (1) are corrected for age, previous radiotherapy, number of brain metastases and previous systemic therapy. Multivariable analyses (2) are corrected for age, number of brain metastases, and SVZ/hippocampal volume.Abbreviations: HVLT-R, Hopkins Verbal Learning Test-Revised; ROCFT, Rey Osterieth Complex Figure Test; TMT, trail making test; VAT, visual association test.

Tables 2 and 3 and Figure 2, along with Figures S3 and S4 display the results of both the univariable and multivariable logistic regression between either mean SVZ or hippocampal dose and change in performance on the memory and information processing speed tasks. A higher mean radiotherapy dose administered to the hippocampi exhibited significant predictive value for declined postradiotherapy performance in the majority of memory tasks. Specifically, in multivariable analyses, changes in HVLT-R immediate recall performance demonstrated β coefficients of −0.787 (*P *= .011, 95% confidence interval [CI] = −1.376 to −0.197) and −0.724 (*P *= .024, 95% CI = −1.348 to −0.100) per Gy on the hippocampi. Similarly, changes in performance on the HVLT-R delayed recall exhibited βs of −0.587 (*P *= .036, 95% CI = −1.133 to −0.042) and −0.574 (*P *= .042, 95% CI = −1.128 to −0.021) per Gy on the hippocampi. Notably, alterations in ROCFT delayed copy performance showed a significant β of −0.700 (*P *= .046, 95% CI = −1.387 to −0.014) per Gy on the hippocampi, but only in the multivariable analysis that controlled for age, previous radiotherapy, number of BMs, and previous systemic therapy. Changes in VAT Immediate Recall performance were not significantly associated with median radiation dose to the hippocampi in either univariable or multivariable analysis. The median dose on the SVZ did not predict changes in memory performance for any of the tasks. Furthermore, no association was observed between changes in information processing speed and radiotherapy dose on the SVZ or hippocampi in either univariable or various multivariable setups.

**Table 2.** Univariable and multivariable linear regression analysis of median radiotherapy dose on the HPC or SVZ on change in performance on memory tasks

	Memory tasks		HVLT-R Immediate	HVLT-R Delayed	HVLT-R Recognition	ROCFT Delayed	VAT Immediate
**Median Hippocampus dose**	Univariable:	*P* value	0.876	0.210	0.585	0.696	0.490
		β	−0.353	−0.240	0.131	−0.096	−0.181
		(95% CI)	(−0.762, 0.055)	(−0.623, 0.142)	(−0.351, 0.612)	(−0.594, 0.401)	(−0.709, 0.347)
	Multivariable (1), corrected for: Age, previous RTP, number of BMs, previous systemic therapy	*P* value	**0.011**	**0.036**	0.383	**0.046**	0.581
		β	**−0.787**	**−0.587**	0.281	**−0.700**	−0.238
		(95.0% CI)	(−1.376, −0.197)	(−1.133, −0.041)	(−0.367, 0.928)	(−1.387, −0.014)	(−1.113, 0.637)
	Multivariable (2), corrected for: Age, number of BMs, HPC-volume	*P* value	**0.024**	**0.042**	0.309	0.059	0.689
		β	**−0.724**	**−0.574**	0.338	−0.678	−0.170
		(95.0% CI)	(−1.348, −0.100)	(−1.128, −0.021)	(−0.329, 1.005)	(−1.384, 0.028)	(−1.032, 0.692)
**Median subven­tricular zone dose**	Univariable:	*P* value	0.559	0.791	0.381	0.674	0.221
		β	−0.121	−0.050	0.205	0.102	−0.302
		(95.0% CI)	(−0.538, 0.296)	(−0.435, 0.334)	(−0.265, 0.675)	(0.386, 0.590)	(−0.795, 0.191)
	Multivariable (1), corrected for: Age, previous RTP, number of BMs, previous systemic therapy	*P* value	0.330	0.412	0.185	0.341	0.124
		β	−0.337	−0.254	0.454	−0.368	−0.612
		(95.0% CI)	(−1.033, 0.358)	(−0.879, 0.370)	(−0.229, 1.137)	(−1.144, 0.409)	(−1.405, 0.180)
	Multivariable (2), corrected for: Age, number of BMs, SVZ volume	*P* value	0.451	0.384	0.115	0.336	0.144
		β	−0.263	−0.266	0.531	−0.368	−0.581
		(95.0% CI)	(−0.964, 0.438)	(−0.882, 0.349)	(−0.137, 1.198)	(−1.136, 0.400)	(−1.373, 0.212)

Abbreviations: BMs, brain metastases; HPC, hippocampus; HVLT-R, Hopkins Verbal Learning Test-Revised; ROCFT, Rey Osterieth Complex Figure Test; RTP, radiotherapy; SVZ, subventricular zone; VAT, visual association test.

Boldfaced values indicate analyses with *P* < .05 and are considered statistically significant.

**Table 3.** Univariable and multivariable linear regression analysis of median radiotherapy dose on the HPC or SVZ on change in performance on information processing speed tasks

	Information processing speed tasks		TMTA	STROOP 1	STROOP 2
**Median Hippocampus dose**	Univariable:	*P* value	0.892	0.880	0.577
		β	0.043	0.052	0.297
		(95.0% CI)	(−0.603, 0.690)	(−0.646, 0.751)	(−0.772, 1.365)
	Multivariable (1), corrected for: Age, previous RTP, number of brain metastases, previous systemic therapy	*P* value	0.748	0.842	0.855
		β	−0.158	−0.102	−0.150
		(95.0% CI)	(−1.155, 0.839)	(−1.141, 0.936)	(−1.809, 1.510)
	Multivariable (2), corrected for: Age, number of brain metastases, HPC-volume	*P* value	0.845	0.524	0.876
		β	−0.100	−0.026	−0.130
		(95.0% CI)	(−1.132, 0.932)	(−1.097, 1.044)	(−1.813, 1.553)
**Median subventricular zone dose**	Univariable:	*P* value	0.620	0.459	0.919
		β	−0.156	−0.251	0.053
		(95.0% CI)	(−0.789, 0.477)	(−0.932, 0.430)	(−1.001, 1.108)
	Multivariable (1), corrected for: Age, previous RTP, number of brain metastases, previous systemic therapy	*P* value	0.192	0.079	0.304
		β	−0.681	−0.942	−0.897
		(95.0% CI)	(−1.723, 0.360)	(−2.002, 0.117)	(−2.648, 0.855)
	Multivariable (2), corrected for: Age, number of brain metastases, SVZ-volume	*P* value	0.356	0.089	0.3782
		β	−0.461	−0.833	−0.746
		(95.0% CI)	(−1.465, 0.543)	(−1.903, 0.237)	(−2.447, 0.956)

Abbreviations:  BMs, brain metastases; HPC, hippocampus; RTP, radiotherapy; SVZ, subventricular zone; TMT, Trail Making Test.

Moreover, no significant correlation was detected between changes in information processing speed and the dosage of radiotherapy administered to the SVZ or hippocampi, as shown by both univariable and multivariable analyses.

## Discussion

Patients with BMs often experience cognitive decline postradiotherapy,^10^ yet the underlying mechanisms remain incompletely understood. In this clinical study, we investigated the impact of radiotherapy dose on neurogenic niches and subsequent cognitive changes 3 months post-SRS, specifically focusing on memory and information processing speed. Our findings indicate that a higher median radiation dose administered to the hippocampi is associated with cognitive decline on memory tasks, but not information processing speed. The median radiotherapy dose to the SVZ did not correlate with declines in either memory or information processing speed performance, underlining the specificity of these associations. Thus, minimizing radiation exposure to the hippocampi, even with SRS, could have potential benefits in preserving memory function in this vulnerable patient population.

Our study provides concrete evidence on the risks associated with radiation exposure to the hippocampi. Specifically, our study showed that for every 1 Gy increase in radiation dose to the hippocampi, there was a noticeable decline in verbal memory performance. This decline ranged from 0.57 to 0.79 points (ie, RCI), depending on the specific memory task. For a population that is already cognitively vulnerable and where the majority already have memory issues prior to SRS,^10^^,^^17^ additional deterioration in memory could have a major impact on their daily lives. The median hippocampal dose in our sample was 89 cGy (IQR = 14-188 cGy), suggesting that incidental high radiation doses to the hippocampi are infrequently administered. Previous studies have shown that reducing the hippocampal dose by more than 50% is achievable with SRS using dose optimization.^33^ Given the low incidence of hippocampal or peri-hippocampal BMs, the subsequent risk of local underdosing due to hippocampal avoidance radiotherapy is deemed minimal.^43^ In line with this, in our study only 2 patients (6%) had hippocampal or peri-hippocampal BMs.

As anticipated based on our previous research,^10^ a substantial proportion of patients exhibited memory deterioration across the various tests 3 months post-SRS. The current findings have linked this decline in memory performance to higher radiotherapy doses to the hippocampi, particularly for tasks involving the learning and delayed recall of verbal and visual information. This finding is consistent with a previous study involving patients with either BMs or glioma, which reported a greater decline in delayed verbal recall 6 months postradiotherapy when the hippocampus was radiated with a higher dose.^44^ This can also explain the lack of predictive value of SVZ radiation dose for memory decline, as the SVZ is likely not specifically involved in memory processes. However, we did not find associations between hippocampal dose and performance on tasks involving learning new associations (VAT immediate recall) or verbal recognition memory (HVLT-R delayed recognition) after 3 months, despite a considerable number of patients showing declined performance. This aligns with previous research indicating that while hippocampal radiation dose affects delayed verbal recall, it does not impact discriminability on recognition tasks in survivors of childhood low-grade glioma.^45^ From a neuropsychological perspective, learning new associations and recognition tasks both involve cued recall, either in forming new associations or assessing familiarity with a cue, while both immediate and delayed recall rely on the free recall of new information. Recognition memory can remain stable in patients with hippocampal damage, likely due to the involvement of non-hippocampal areas.^46^^,^^47^ This reliance on other brain regions could explain the discrepancy between different memory tasks observed in our study. These results highlight the importance of sparing the hippocampus to protect memory function while also suggesting that non-hippocampal areas play a crucial role in the complex processes of learning and memory, warranting further investigation. The timing of these effects, potential for neural plasticity and repair over longer periods, and whether cognitive changes stem from localized damage or broader network disruptions are still open questions. Further clinical and preclinical research is needed to elucidate these aspects.

In addition, no significant correlation was found between radiotherapy dose to the SVZ and memory decline. Moreover, radiation to either region was not associated with change in information processing speed. Similar nonsignificant associations for attention performance and the hippocampal radiation volume were observed in a study with childhood survivors of low-grade glioma.^45^ The most likely explanation is that while the SVZ plays a pivotal role in neurogenesis and brain repair mechanisms in adults^19^ and survival,^25^ it cannot be linked to specific neurocognitive processes, like memory performance. Moreover, information processing speed may be more reliant on the functional integrity of frontoparietal white matter connections than a single location such as the SVZ or hippocampus.^48^ Future research should continue to explore the specificity of these relationships across various cognitive functions and brain regions, and on timing of occurrence after radiotherapy.

We used linear regression to examine the relationship between radiotherapy dose to the hippocampi and cognitive changes. This approach allowed us to investigate the continuous nature of the dose-response relationship, rather than identifying a specific dose threshold or cut-off point. Our findings suggest a proportional relationship between dose and cognitive decline, particularly in memory function. Assuming a cause-and-effect relationship, the results suggest a specific vulnerability of the hippocampus that extends beyond neurogenesis, as damage to the SVZ was not related to memory performance. This implies that other forms of damage may contribute to the overall reduced functionality of the hippocampus, highlighting the need for a comprehensive approach to minimize radiation exposure to this critical region. While studies comparing hippocampal-avoidance radiotherapy (HA-RT) with conventional approaches have yielded mixed results, our focus on SRS provides a unique perspective.^49^ Our results may reflect the interconnectedness of brain networks; as sparing the hippocampus alone while damaging its connections (as in HA-WBRT) might not sufficiently preserve cognitive function. Unlike traditional avoidance studies that might establish strict dose constraints, our findings support a more nuanced treatment planning approach, where even small reductions in hippocampal dose could yield cognitive benefits. This highlights the need for tailored strategies in different radiotherapy modalities and the complexity of radiation’s impact on cognitive outcomes.

Multiple factors could have contributed to the observed post-SRS cognitive decline. For example, prolonged systemic chemotherapy has been linked to decreased neurogenesis in the hippocampus and reduced learning abilities in rats.^50^ Therefore, these factors are crucial to correct for in our analysis, as likely reflected in the significant multivariate results obtained after accounting for these variables. Additionally, we specifically investigated cognitive decline 3 months after radiotherapy, which coincides with the period when systemic therapy is often reinitiated. Previous research has shown both improvements and further declines post-SRS in cognitive performance beyond the 3-month timepoint.^9^^,^^10^ Therefore, it would be interesting to investigate the long-term cognitive performance scores in relation to both radiotherapy and systemic therapy. We included a diverse group of patients with BMs, encompassing different primary tumors, a wide age range, and an equal gender distribution, reflecting a broad spectrum of clinical practices and patient demographics, enhancing generalizability. This diversity enhances the applicability of our findings to a wider patient population. Ideally, we would have liked a larger sample size with a more even distribution between the groups of patients with lesions in contact with the SVZ or hippocampi and those without. This would have enhanced the statistical power of our analysis and enabled subgroup analyses, including considerations such as the type of primary cancer and the number of BMs. Although the uneven distribution between patient groups limited statistical power and subgroup analyses (eg, by primary tumor type or number of BMs), despite the small sample size, our study confirmed the hypothesis of a specific link between hippocampal dose and memory processes, highlighting the value of our findings. Excluding patients with WBRT not only minimized outliers in radiotherapy dose, but additionally made our results more generalizable to the Dutch BMs population, as SRS is the treatment of choice for up to 10 BMs according to the consensus guidelines in the Netherlands.^51^ The use of a specifically designed atlases allowed for accurate delineation of regions of interest^24^^,^^40^ and the absence of a priori selection based on preradiotherapy cognitive functioning further strengthen the potential clinical significance and impact of the study.

This study has some limitations. The relatively small sample size and the heterogeneity within the sample limited the possibility for subgroup analyses and reduced statistical power, necessitating caution in interpreting the results. Due to these constraints, we had to limit the number of covariates included in the analyses, which meant that potentially relevant factors such as mood could not be accounted for as corrections. While mood may influence certain cognitive outcomes, such as processing speed, the evidence for a consistent association in this population is mixed. Future studies with larger and more homogenous samples should include broader covariate sets, including mood assessments, to clarify these relationships. In many studies of patients with BMs, roughly 40% to 50% of participants are unavailable for 3-month to 4-month follow-up, mainly due to death, clinical deterioration, or logistical challenges. This attrition rate is consistent with what we observed in our study and reflects the inherent challenges of longitudinal assessments in this vulnerable population. Last, although the Rey Complex Figure Test does not have a universally accepted alternate form, we mitigated practice-effects by using the Taylor Complex Figure as a parallel version alongside the use of the RCI. This approach provides a conservative assessment of cognitive change. Future studies should consider additional visuospatial memory measures with standardized alternate forms to further address radiation-related effects.

## Conclusion

The treatment of BMs remains a delicate balance between maximizing disease control and minimizing side effects. Our study underscores the potential benefits of minimizing radiotherapy dose to the hippocampi to protect cognitive functioning, particularly in the current treatment era. As patients with BMs are living longer, they face an increased likelihood of experiencing posttreatment cognitive deficits and must cope with these impairments for extended periods. These findings highlight the potential for improving long-term outcomes for patients with BMs through targeted radiation-sparing techniques.

## Supplementary Material

npag001_Supplementary_Data

## Data Availability

Upon completion of the APRICOT and COIMBRA trial, data can be made available pending a formal research proposal.

## Lay Summary

Patients with brain metastases, cancer that has spread to the brain, often receive precise radiotherapy called stereotactic radiosurgery. However, even this treatment can affect healthy brain areas important for memory and thinking, such as the hippocampus. Our study examined whether radiation to specific regions was linked to changes in memory and processing speed 3 months after treatment. We found that higher radiation doses to the hippocampus were associated with memory decline, but not with processing speed or other brain areas. These findings suggest protecting the hippocampus during stereotactic radiosurgery for brain metastases may help preserve memory, although more research is needed.
