# Supplementary material for: Negative impact of hippocampal radiotherapy dose on memory function in patients with brain metastases
Source: Neurooncol Pract. 2026 Jan 10;13(3):506–17. doi: 10.1093/nop/npag001 (PMC13161910; doi:10.1093/nop/npag001)
Supplement: npag001_Supplementary_Data [file npag001_supplementary_data.docx]

# Supplementary materials

**Supplementary Table S1:** Description of educational levels according to the Verhage classification system (adapted from Verhage, 1964).^1^

| **Level** | **Verhage Categories** |
| --- | --- |
| Low | 1. Less than 6 years of primary education |
|  | 2. Finished primary education |
|  | 3. Primary education and less than 2 years of low-level secondary education |
|  | 4. Finished low-level secondary education |
| Middle | 5. Finished average-level secondary education |
| High | 6. Finished high-level secondary education |
|  | 7. University degree |


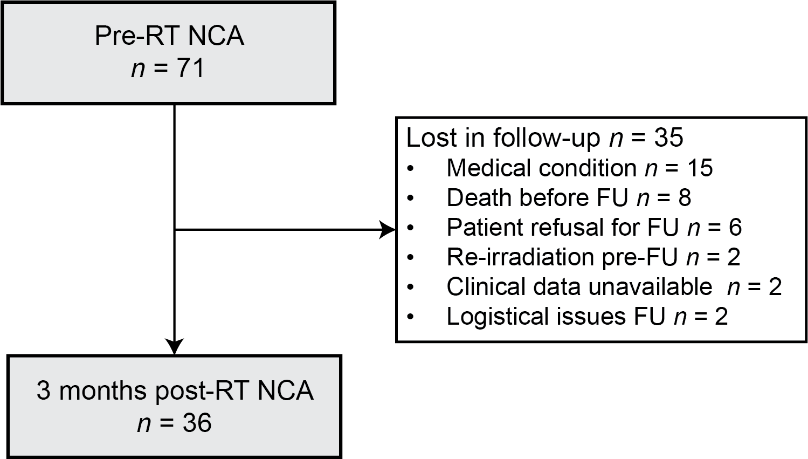


**Supplementary Figure S1**. Flow-chart of the patients completing the pre-radiotherapy and 3 months post-radiotherapy neurocognitive assessment including reasons for patients lost in follow-up. *Abbreviations: NCA, neurocognitive assessment; RT, radiotherapy;*


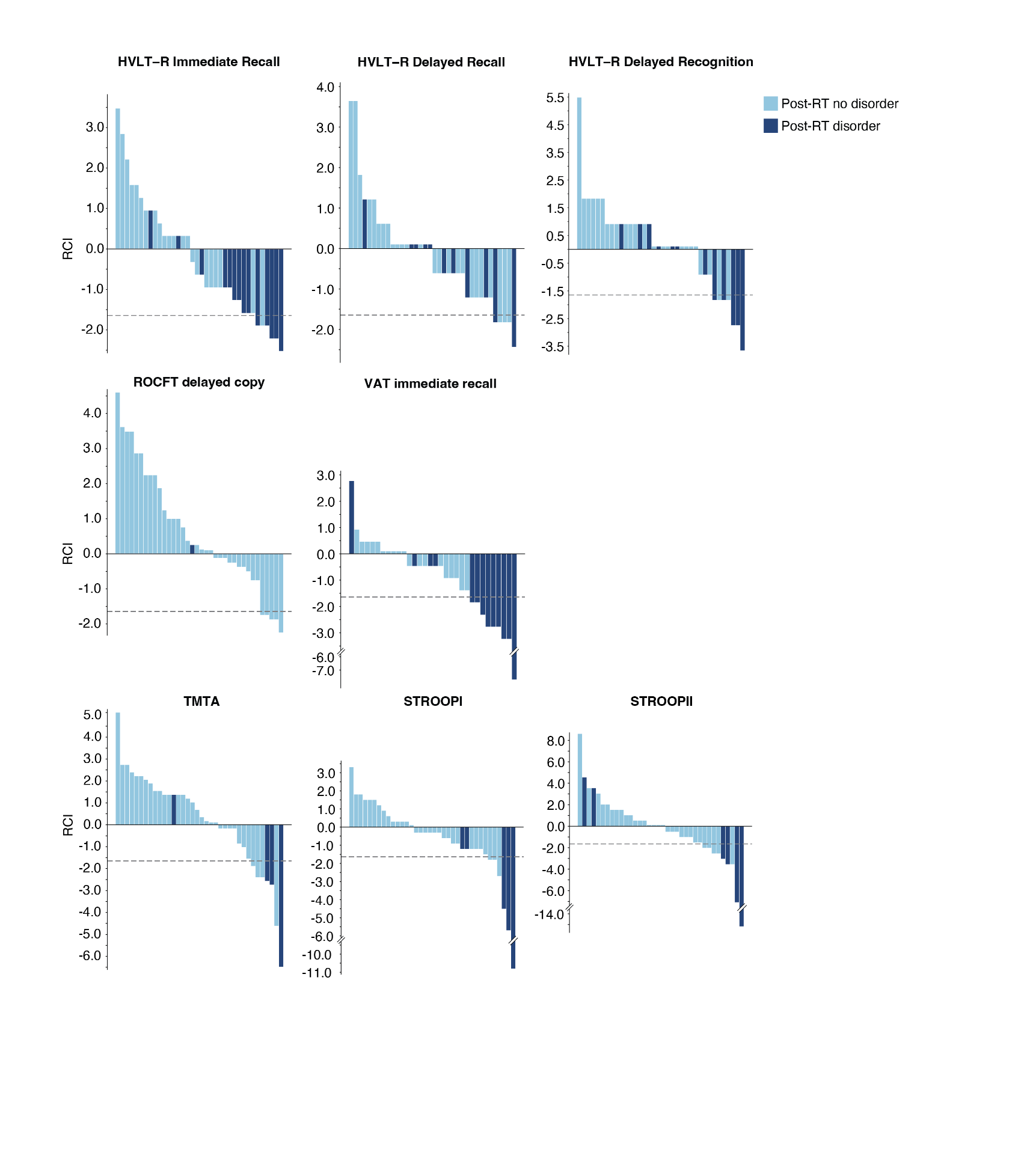


**Supplementary Figure S2**. Waterfall plots displaying reliable change indices for the various cognitive tests. Each bar represents an individual, with colors denoting whether the patient's z-score falls below or above -1.5, indicating cognitive disorder (dark blue) or no disorder (light blue), respectively. A dotted grey line marks an RCI of -1.645, typically used to indicate significant change.

**Supplementary Figure S3.** The regression plots showing the univariable regression between dose in either hippocampi or SVZ and the RCI for the different memory tasks


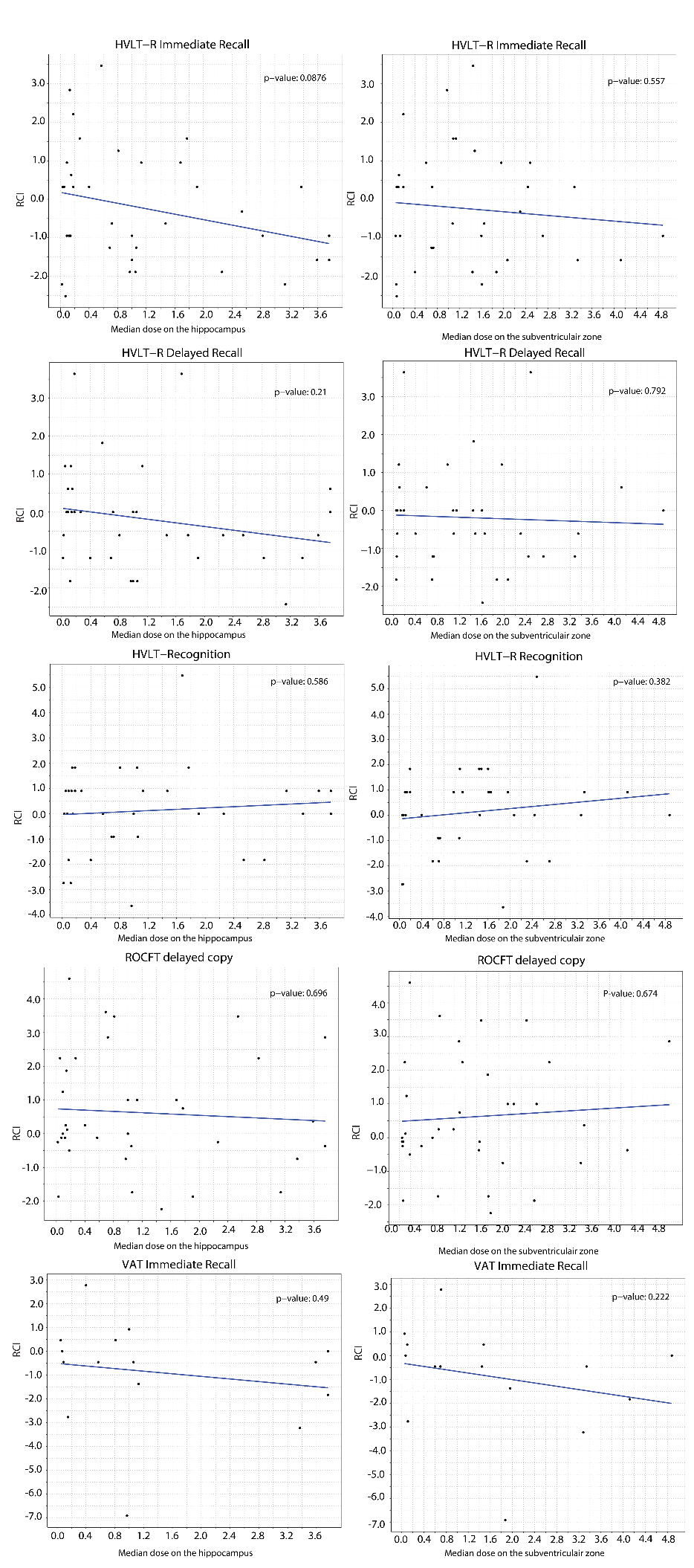


**Supplementary Figure S4.** The regression plots showing the univariable regression between dose in either hippocampi or SVZ and the RCI for the information processing speed


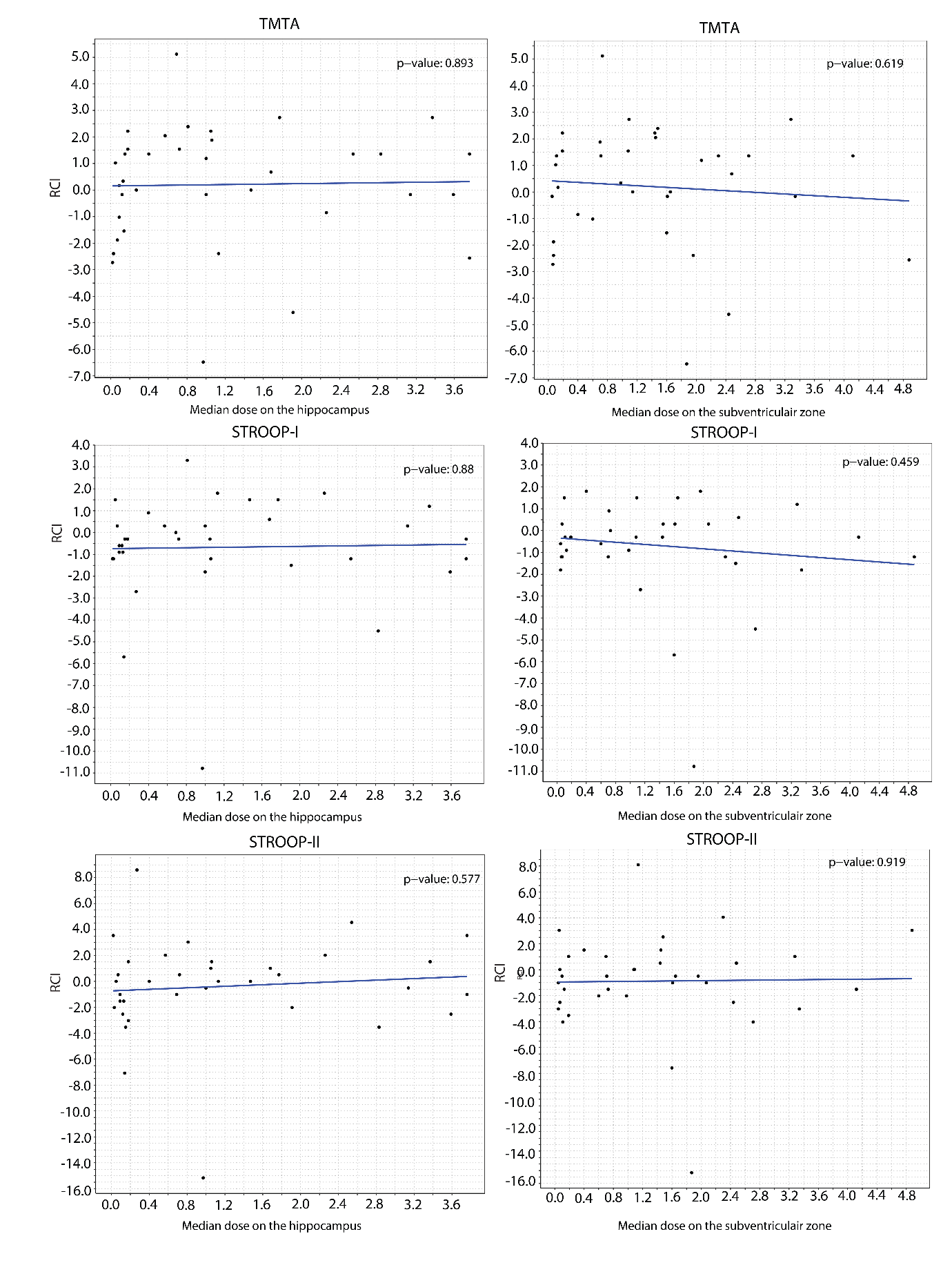


1. Verhage F. Intelligentie en leeftijd bij volwassenen en bejaarden. Koninklijke van Gorcum. 1964;
